# On the Influence Which Water Exerts on the Physical Properties of Various Solid Azotated (Animal) Substances

**Published:** 1821-12

**Authors:** 

**Affiliations:** Professor of Chemistry, &c.


### *On the Influence which Water exerts on the Physical Properties of various solid azotated (Animal) Substances.* By Mr. Chevreul, Professor of Chemistry, &c.

[Concluded from page 468.]

PART II.

IT has been seen, from the observations contained in the first part of the present essay, that the tendons, the yellow tissue, the coagulated albumen, fibrine, cartilage, and the dry cornea, bear, externally, so close a resemblance to each other, that they can with difficulty be distinguished. If, however, they be immersed in water, each of these substances absorbs a certain quantity of that liquid, and acquires, at the same time, properties of a very distinct character. Thus, a tendon becomes supple and *satiné;* the yellow tissue acquires a considerable degree of elasticity; coagulated albumen assumes the aspect of the white of a boiled egg; fibrine becomes white and elastic; the cartilage white also, and flexible; and, lastly, the opaque cornea resumes the same aspect by which it is distinguished in the living animal. We have attributed these properties to the presence of water, because we invariably find them, when organic substances combine with that liquid; whereas, they disappear on those substances becoming dry. Not only can water be separated from these organic substances by simple exposure to the air, and, *a fortiori*, by means of the dry and exhausted receiver; but it is also possible to subtract a considerable portion of that fluid, by submitting the substances containing it to the action of the press, after having enveloped them within several folds of blotting paper, so as to prevent evaporation. It is worthy of remark, that the quantity of water thus abstracted from the tendon and yellow tissue is sufficiently large to render them transparent, while they at the same time lose, the first its flexibility, and the second its elasticity.

Some tendons, which, when exposed to the air, would have lost 53 per cent. of water, having been subjected to the action of a small press, lost 37.6; while the yellow tissue, which by exposure to air would have lost 48.2 parts of water, lost 35 of it when submitted to pressure.

The forces which can act on the water contained in recent organic substances, are, on the one hand, affinity, and the action of the particles of that fluid on each other, (or, in other words, cohesion,) on the other hand. Let us now endeavour to ascertain how far these two forces may exert their influence in the production of the phenomena we have recorded.

There is no doubt that a certain quantity of water is, from affinity, intimately combined with organic substances, since all these substances, however properly dried, are more or less hygrometric; and every one knows that a body which condenses the vapours of water, is enabled to do so by virtue of chemical action. Yet, in these same substances, when saturated with water, there may be another portion of that fluid present, which is not subject to the laws of affinity. We may, in reality, admit that the quantity of water in question penetrates into the organic tissue, as a necessary consequence of the cohesion of its particles. Thus, in a moist sponge, the water is subject to its own affinity for the organic tissue with which it is in contact, and to the cohesion of its own particles, at one and the same time; for, as affinity acts only when bodies are in apparent contact, it would be as absurd to suppose that the particles of water, placed in the centre of the large interstices of the sponge, obey the impulse of that force, as to consider that the central strata of a liquid, raised within a capillary tube, are susceptible of receiving any chemical influence from the materials of which the tube itself is composed.

These facts being thus far explained, it will naturally be asked, whether there be not found, in the substances mentioned in the present memoir, a given proportion of water, which is independent of the laws of affinity. While we admit our insufficiency to resolve this question, we cannot help observing, that the opacity of these substances would lead us to believe in the affirmative; for they are transparent as long as they retain the quantity of water only which is combined with them by affinity, but lose their transparency the moment they absorb an additional portion of water, by the assistance of which they seem to acquire flexibility. Besides, the impossibility of imparting any degree either of flexibility or elasticity to these same substances, when in a dried state, by immersion in oil, alcohol, or other liquids, (which one would be disposed to consider as more likely to penetrate their most intimate texture,) would appear to indicate that a chemical action obtains between the organic tissues and that portion of water which so materially modifies their physical properties.

Whatever be the opinion entertained respecting the actual state of that portion of water which is constantly met with in fresh organic tissues, the observations contained in the present memoir will not be found altogether devoid of interest: first, because of the great influence which an additional quantity of water exerts on animal substances already containing a certain portion of that fluid in combination; and, secondly, in consequence of the application that may be made of those facts to animal physiology.

It is a well-known fact, that the organic tissues of animals are more tender and more gelatinous at an early than at an advanced age; and it is scarcely to be doubted that this fact depends on the greater proportions of water which those tissues are susceptible of receiving in young than in old animals. It will probably be found, with reference to the particular fact which forms the object of this paper, that, in studying the same tissue in the same animal, taken at different ages, and in animals of different species, more satisfactory conclusions will be obtained than have hitherto been drawn respecting the anatomy and physiology of those organs. It will be curious to ascertain, if possible, by means of microscopical examinations, whether any difference of structure exist in those tissues which follow different laws in their combinations with water; and, *vice versa*, how far those tissues which seem to be similarly affected by that fluid are alike in their texture.

When the facts mentioned in this paper are taken into consideration, a positive proof is obtained that water, independently of its use in the animal economy as an excipient of the blood and humours, or for the purpose of tempering the effects of excessive heat, to which animals may be exposed, is one of the principles which has the most influence on the existence of animated beings, in consequence of the kind of action it exerts on their organic tissues.

Let us, in fact, reflect, that the tendons, the yellow tissue, and the muscles, of all which the essential principle is fibrine, are the organs which act the most conspicuous part in the movements of animals; and that, without the presence of that quantity of water, of which mention has so often been made in the present memoir, they are incapable of performing that duty; and the importance of water in animal substances will readily be admitted.

It is not difficult to understand how numerous must be the disorders in the exercise of the functions of life which must ensue, where the animal loses too large a quantity of water through the surface of the body. Saussure was perfectly correct in attributing the vesications and cracking of the skin, fissures, and chilblains, to which a man is subject when he ascends very high mountains, to the excessive dryness of the atmosphere in those regions. Our own observations confirm the statement of the philosopher of Geneva; and we hesitate not to add, that the noxious effects of dryness easily extend from the surface to the internal organs of the animal system. This view of the subject coincides with the experiments made by Dr. Edwards, who has ascertained that one of the principal causes of death in fish out of the water, is the diminution in the weight of their bodies consequent on the loss of a certain quantity of water by transpiration.

